# Hemostatis Analyzer-Supported Hemotherapy Algorithm in Cardiac Surgery: Protocol for a Randomized Controlled Monocentric Trial

**DOI:** 10.2196/17206

**Published:** 2020-04-21

**Authors:** Sophie Michel, Florian Piekarski, Jan-Hendrik Fischer, Vanessa Hettler, Elisabeth Hannah Adam, Lars Holzer, Gösta Lotz, Thomas Walther, Kai Zacharowski, Florian Jürgen Raimann

**Affiliations:** 1 Department of Anaesthesiology, Intensive Care Medicine and Pain Therapy University Hospital Frankfurt Goethe University Frankfurt am Main Germany

**Keywords:** Quantra, cardiothoracic surgery, bypass, coagulopathy, point of care, algorithm, rotational thromboelastometry, Multiplate

## Abstract

**Background:**

Point of care devices for performing targeted coagulation substitution in patients who are bleeding have become increasingly important in recent years. New on the market is the Quantra. It is a device that uses sonorheometry, a sonic estimation of elasticity via resonance, which is a novel ultrasound-based technology that measures viscoelastic properties of whole blood. Several studies have already shown the comparability of the Quantra with devices already established on the market, such as the rotational thromboelastometry (ROTEM) device.

**Objective:**

In contrast to existing studies, this study is the first prospective interventional study using this new system in a cardiac surgical patient cohort. We will investigate the noninferiority between an already existing coagulation algorithm based on the ROTEM/Multiplate system and a new algorithm based on the Quantra system for the treatment of coagulopathic cardiac surgical patients.

**Methods:**

The study is divided into two phases. In an initial observation phase, whole blood samples of 20 patients obtained at three defined time points (prior to surgery, after completion of cardiopulmonary bypass, and on arrival in the intensive care unit) will be analyzed using both the ROTEM/Multiplate and Quantra systems. The obtained threshold values will be used to develop a novel algorithm for hemotherapy. In a second intervention phase, the new algorithm will be tested for noninferiority against an algorithm used routinely for years in our department.

**Results:**

The main objective of the examination is the cumulative loss of blood within 24 hours after surgery. Statistical calculations based on the literature and in-house data suggest that the new algorithm is not inferior if the difference in cumulative blood loss is <150 mL/24 hours.

**Conclusions:**

Because of the comparability of the Quantra sonorheometry system with the ROTEM measurement methods, the existing hemotherapy treatment algorithm can be adapted to the Quantra device with proof of noninferiority.

**Trial Registration:**

ClinicalTrials.gov NCT03902275; https://clinicaltrials.gov/ct2/show/NCT03902275

**International Registered Report Identifier (IRRID):**

DERR1-10.2196/17206

## Introduction

### Background

A targeted coagulation therapy during intra- and postoperative care for cardiac surgical patients needs an accurate knowledge of their hemostatic conditions. For the purpose of coagulation diagnosis, many institutions take blood samples and send them to the central clinical chemist. After analysis and validation, results are transmitted electronically, which might prolong the time required to derive therapeutic interventions.

In recent years, point-of-care testing (POCT) devices for the diagnosis of patients who are coagulopathic have become increasingly important. In a neurosurgical trial, Beynon et al [1] showed that the use of POCT markedly reduced the time to receive clotting parameter results in comparison to conventional laboratory analyses. Moreover, the quality of the results (eg, the international normalized ratio and conventional laboratory results) in POCT also showed a high correlation in values [2].

POCT devices have been routinely used for guiding intra- and postoperative targeted coagulation therapy for years in our clinic. The devices in use include the rotational thromboelastometry (ROTEM) delta and Multiplate. For the purpose of analysis, a whole blood sample is pipetted and mixed with test reagents. Depending on the selected reagents, different steps of the coagulation cascade can be evaluated, and, according to each parameter result, an appropriate therapy can be derived.

A coagulation algorithm based on those measurements has been developed in our clinic, and a modified version for cardiac surgery has been used successfully for years in perioperative coagulation management [3]. The algorithm requires additional information on the platelet function, which is also carried out as a standard practice on the bedside using the Multiplate device.

The Quantra Analyzer system from HemoSonics, a new system for hemostasis analysis, has recently become available on the market [4]. The Quantra system also allows the analysis of a whole blood sample on the bedside. Moreover, due to the fully sealed cartridge system of the Quantra, pipetting of a whole blood sample is no longer necessary and hence avoids the time-consuming and potentially error-prone procedure. In addition, the risk of infection for the examiner is smaller. An evaluation of the platelet activity for the Quantra device system in a cardiac surgical patient trial, [5] as well as the comparability procedures with ROTEM measures has already been assessed [5,6].

### Objectives

In this first interventional study, our main objective is to show the noninferiority of a new Quantra-based hemotherapy algorithm in comparison to an existing algorithm based on the ROTEM delta and Multiplate used in our clinical routine. We expect comparable results in the effects of stabilizing coagulation after cardiosurgical interventions and postoperative blood loss.

## Methods

### Materials

To guarantee effective coagulation management during cardiosurgical interventions, we already use a coagulation algorithm adapted to the results of point-of-care measurements for coagulation and platelet function.

In our clinic the ROTEM device is used to analyze clotting time, clot formation, clot stiffness, and the dissolution of the clot (fibrinolysis). In addition to ROTEM, we use the Multiplate system to obtain information about the platelet function and to detect a possible presence of adenosine-diphosphate (ADP) antagonists like Clopidogrel (ADPtest) or cyclooxygenase-inhibitors (arachiconic acid test). Based on the results of the ROTEM and Multiplate system, we administer a systematic coagulation therapy in accordance with an already existing algorithm.

**Figure 1:**
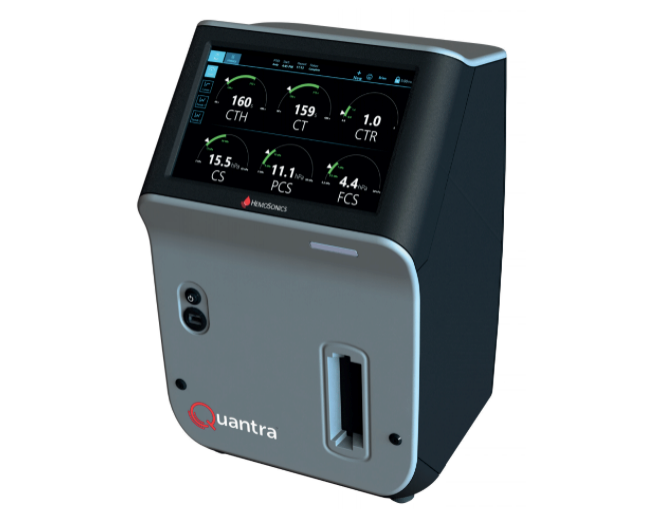
Quantra Analyzer. CS: clot stiffness; CT: clot time; CTH: heparinase clot time; CTR: clot time ratio; FCS: fibrinogen contributions to clot stiffness; PCS: platelet contributions to clot stiffness.

**Figure 2:**
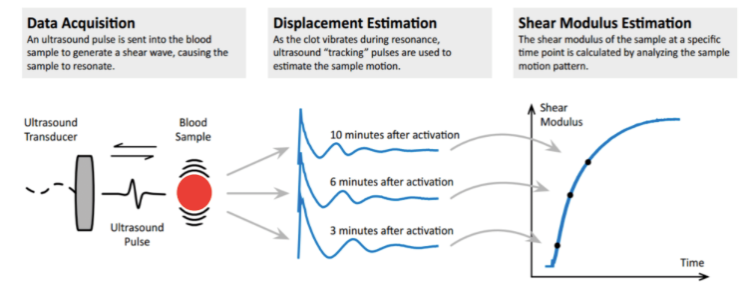
Principle of SEER Sonorheometry.

A new system, the Quantra Hemostasis Analyzer (Figure 1) is based on sonic estimation of elasticity via resonance. Sonorheometric technology uses high-frequency ultrasound for analyzing changes in viscoelastic properties of whole blood samples (Figure 2). For this purpose, a cartridge with four channels, containing different reagents in every channel, is used (see Table 1).

**Table 1:** Design and assignment of the individual channels of the measuring cartridge.

Cartridge channel	Reagents	Possible measurements
1	Kaolin (intrinsic coagulation)	Clot time
2	Kaolin Heparinase (heparin neutralization)	Clot time with heparinase
3	Thromboplastin (extrinsic coagulation, tissue factor activation) Polybrene (heparin neutralization)	Clot stiffness
4	Thromboplastin (tissue factor activation) Polybrene (heparin neutralization) Abciximab (platelet inhibition)	Fibrinogen contribution
1-2	N/A^a^	Clot time ratio
3-4	N/A	Platelet contribution

^a^N/A: not applicable.

**Figure 3:**
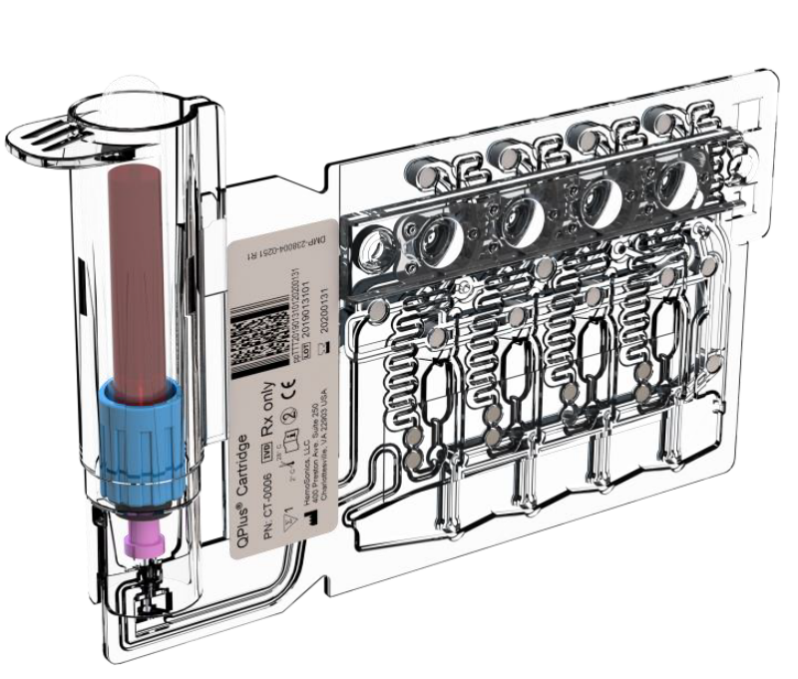
QPlus cartridge.

The user inserts a whole blood sample (collected in a 2.7 ml sodium citrate tube) into the cartridge (QPlus cartridge, Figure 3). The four tests run simultaneously and in a self-contained system. An ultrasound pulse, which is transmitted into the blood sample chamber, generates a shear wave as it clots. Thus, the resonance generated by the displacement of a developing clot in the blood sample is recorded. A series of ultrasound pulses is necessary to estimate the sample’s motion. The generated shear wave modulus (stiffness) of the blood is then measured at precise time points [7].

The Quantra system has already been used for evaluation of hemostatic function in critical care and operative settings, and was compared with laboratory parameters and ROTEM [5,7].

### Study Phases

**Figure 4:**
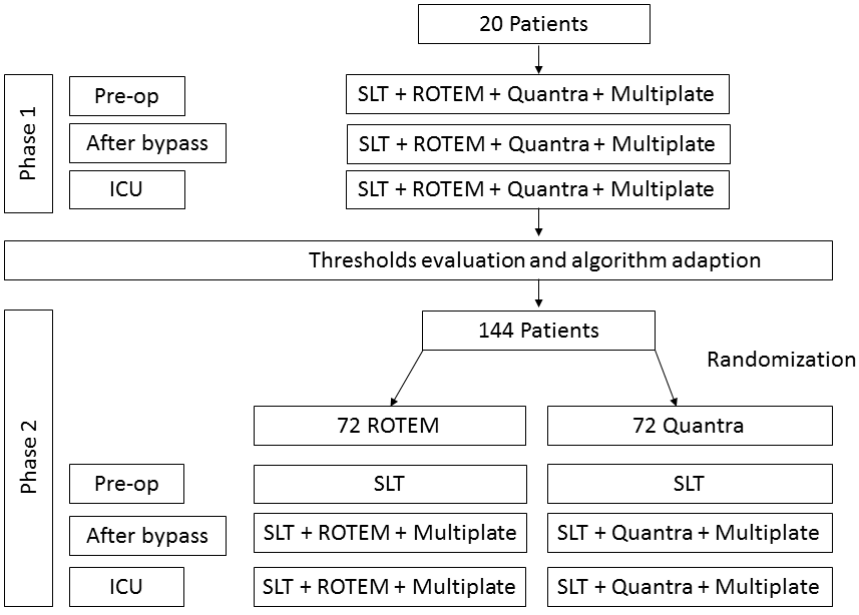
Study flow chart. ICU: intensive care unit; ROTEM: rotational thromboelastometry; SLT: standard laboratory test.

The study is divided into two phases (see Figure 4). Recruiting started in September 2019. It is planned to include 20 patients in phase one and 144 patients in phase two. The estimated end of the study is calculated to be December 2020.

### Patient Population

Inclusion criteria will be patients 18 years of age or older undergoing elective cardiac surgery on cardiopulmonary bypass.

Patients without written consent or missing data will be excluded.

### Observational Phase

In an initial phase, 20 patients undergoing elective cardiac surgery will be examined at the following three time points, using the ROTEM and Multiplate, as well as the Quantra.

1. Directly prior to surgery
2. After completion of the bypass and before reversal of heparin with protamine
3. On arrival in the intensive care unit

The data obtained will be used to evaluate results and threshold values for therapy. Those results will serve to review and adapt the already existing algorithm used in our clinic. All therapeutic decisions at this time will still be made based on the existing coagulation algorithm using the ROTEM and Multiplate results.

### Interventional Phase

After adapting the existing algorithm to the Quantra POCT results, the second phase will follow to evaluate the use of the new algorithm. For this purpose, 144 patients will be randomized into two groups. Patients of the Quantra group (QG) will be treated according to the new algorithm, while the comparison, or standard group (SG), will be treated using the existing algorithm. All relevant data will be entered in a case report form. Table 2 gives an overview of the tests within the various phases of the study compared with the measurements commonly used in our clinic before, during, and after surgery.

**Table 2:** Planned tests during the study compared to the previous standard in our clinic.

Phase		Standard care	Study procedures	
			Quantra group	Standard group
**Phase 1**				
	Presurgery	Standard laboratory test^a^	Standard laboratory test Quantra/ROTEM^b^/Multiplate	Standard laboratory test Quantra/ROTEM/Multiplate
	During surgery	ROTEM/Multiplate only when bypass >120 min	Standard laboratory test Quantra/ROTEM/Multiplate	Standard laboratory test Quantra/ROTEM/Multiplate
	Postsurgery	Standard laboratory test ROTEM/Multiplate only if persistent bleeding	Standard laboratory test Quantra/ROTEM/Multiplate	Standard laboratory test Quantra/ROTEM/Multiplate
**Phase 2**				
	Presurgery	Standard laboratory test	Standard laboratory test	Standard laboratory test
	During surgery	ROTEM/Multiplate only when bypass >120 min	Standard laboratory test Quantra/Multiplate	Standard laboratory test ROTEM/Multiplate
	Postsurgery	Standard laboratory test ROTEM/Multiplate only in case of persistent bleeding	Standard laboratory test Quantra/Multiplate	Standard laboratory test ROTEM/Multiplate

^a^Standard laboratory tests include hemoglobin, thrombocytes, international normalized ratio, Quick test, activated partial thromboplastin time, antithrombine III, fibrinogen, creatinine, and electrolytes.

^b^ROTEM: rotational thromboelastometry.

### Statistical Analysis

As this is a pilot study, sample size was calculated based on the existing literature and our own clinical data. Our own data suggest that moderate blood loss of 650 mL is expected within 24 hours after surgery.

### Sample Size Consideration

To determine the required number of cases for a statistical power of 80%, it was assumed that the blood loss in both groups would be normally distributed. In-house data shows that blood loss is, for obvious reasons, not normally distributed, and a difference of 150 mL corresponds to a Mann-Whitney estimator of 0.363. Therefore, the one-sided noninferiority test requires a total of 140 evaluable patients (70 in each group) to obtain a statistical power of 80%. To compensate for 2 patients per group being lost to follow-up each groups’ target size is 72 patients. This results in a total population of 144 patients.

### Primary Outcome Analysis

A one-sided significance level of alpha 2.5% will be used in proving the noninferiority of the QG compared to the SG. The noninferiority limit has been set to 150 mL. For testing, a nonparametric Wilcoxon-Mann-Whitney U test will be used for one-sided tests of superiority. In the initial Quantra group 150 mL will be subtracted from the primary target test, as this corresponds to a nonparametric variant of a usual noninferiority test with the Wilcoxon-Mann-Whitney U test.

### Conversions

Quantra clot stiffness values are expressed in hectopascals, whereas corresponding ROTEM values are expressed as an amplitude (A) in mm. The relationship between A in mm and shear modulus (G) in pascals is not linear. For a proper comparison, the ROTEM A (mm) needs to be converted to pascals by using the following formula: G (pascals) = (500 x A) / (100 – A), as described by Solomon C et al [8].

## Results

The main and secondary outcomes are shown in Textbox 1.

The primary outcome will be the cumulative blood loss after cardiac surgery (coronary bypass, valve, and aortic operations) within 24 hours.

**Main and Secondary Outcomes.**

**Primary outcome**

- Cumulative blood loss after cardiac surgery (24 hours)

**Secondary outcomes**

- Correlation between laboratory and point-of-care testing results (Quick test, international normalized ratio, activated partial thromboplastin time, fibrinogen)
- Platelet function with Multiplate-Analysis
- Time until results are obtained (ie, time until therapy can be established)
- Use of blood products (packed red blood cells, fresh frozen plasma, platelets)
- Thromboembolic events
- Comorbidities
- Type of surgery procedure
- Demographic data
- Use of procoagulants (fibrinogen, desmopressin, tranexamic acid, prothrombin complex concentrate, recombinant enabled factor VIIa, calcium)

We expect to receive comparable results regarding the named main and secondary outcomes, both in the SG as well as in the QG. According to this assumption, we want to show the noninferiority of a Quantra-based hemotherapy algorithm compared to the ROTEM and Multiplate system.

This study is approved by the local ethical committee (ID: 42/19) and was started in September 2019. Results will be published soon after completion.

## Discussion

This is the first prospective interventional study comparing the Quantra system with the ROTEM and Multiplate system regarding blood loss in 24 hours after implementing an adapted coagulation algorithm for cardiac surgical patients. Huffmeyer et al [5] showed a significant correlation between the Quantra system and the ROTEM and Multiplate system with respect to clot stiffness and fibrinogen contribution [5].

The focus of our study is whether a Quantra-based hemotherapy algorithm is noninferior to the ROTEM and Multiplate-based algorithm we already use in our clinical routine.

There are some aspects of the Quantra system that could prove beneficial. First, there is no need for pipetting reagents to the full blood sample. Accordingly, we expect less erroneous measurements due to inaccurate pipetting.

Both the accuracy of the results and the time until results are available correlate with the skills of the investigator. We expect the Quantra system to be easy to use and interpret. There will be no extensive training required.

Second, the Quantra Analyzer is a closed system, which minimizes the probability of contamination. In comparison, the ROTEM and Multiplate is an open system and possibly more sensitive to contamination.

## Abbreviations

A: amplitude
ADP: adenosine-diphosphate
G: shear modulus
POCT: point-of-care testing
QG: Quantra group
ROTEM: rotational thromboelastometry
SG: standard group
